# Quantitative study of the ossification centers of the body of sphenoid bone in the human fetus

**DOI:** 10.1038/s41598-024-64550-2

**Published:** 2024-06-12

**Authors:** Magdalena Grzonkowska, Mariusz Baumgart, Michał Szpinda

**Affiliations:** Department of Normal Anatomy, The Ludwik Rydygier Collegium Medicum in Bydgoszcz, The Nicolaus Copernicus University in Toruń, Łukasiewicza 1 Street, 85-821 Bydgoszcz, Poland

**Keywords:** Sphenoid bone, Bone development, Presphenoid ossification center, Postsphenoid ossification center, Osteogenesis, Fetal development, Musculoskeletal system, Bone development, Orthopaedics

## Abstract

The aim of the present study was to examine the growth dynamics of the two ossification centers of the body of sphenoid bone in the human fetus, based on their linear, planar and volumetric parameters. The examinations were carried out on 37 human fetuses of both sexes aged 18–30 weeks of gestation, which had been preserved in 10% neutral formalin solution. Using CT, digital image analysis software, 3D reconstruction and statistical methods, we evaluated the size of the presphenoid and postsphenoid ossification centers. The presphenoid ossification center grew proportionately in sagittal diameter, projection surface area and volume, and logarithmically in transverse diameter. The postsphenoid ossification center increased logarithmically in sagittal diameter, transverse diameter and projection surface area, while its volumetric growth followed proportionately. The numerical findings of the presphenoid and postsphenoid ossification centers may be considered age-specific reference values of potential relevance in monitoring the normal fetal growth and screening for congenital disorders in the fetus. The obtained results may contribute to a better understanding of the growing fetal skeleton, bringing new numerical information regarding its diagnosis and development.

## Introduction

The skeletal system of the human fetus develops during the early stages of gestation. Due to its early growth and high dynamics the skeleton may successfully be monitored in utero using ultrasound (US), computed tomography (CT) and magnetic resonance imaging (MRI). High-resolution echographic and tomographic imaging enables a precise evaluation of ossification centers, thus facilitating the assessment of fetal development and the early detection of skeletal system disorders^1^. CT is considered the most effective technique for a comprehensive assessment of the development of the cranial base, completed by MRI in detecting and identifying cranial base pathologies^2^.

The formation and consecutive growth of the cranial base are strongly associated with the development of the brain. By providing support to various parts of the brain, the cranial base permits and influences its development. Many congenital anomalies of the brain correspond to malformations of the cranial base, such as platybasia, Chiari malformation and agenesis of the corpus callosum. Therefore, a detailed analysis of the development of individual bones in the cranial base is not only important from basic science perspectives, but also relevant because of the complex pattern of bone ossification and its strong relationship with the brain development^3,4^.

The development of the cranial base takes significantly more time compared to that of the viscerocranium or the calvaria. While the calvarian bones rapidly expand in parallel with the external growth of the brain, the cranial base displays slower growth due to the relatively languid development of the basal part of the brain. Furthermore, the early development of the cranial nerves and blood vessels may also influence the ossification process of the cranial base^3,4^.

The sphenoid bone is the most complex structure of the cranium. Due to its very central position in the cranial base and seven components, the sphenoid bone is contributable to all internal (anterior, middle and posterior cranial fossae) and external (temporal, infratemporal and pterygopalatine fossae, orbit and nasal cavity) topographical elements. The sphenoid bone comprises the body of sphenoid bone and the three pairs of processes, called the lesser wings, greater wings and pterygoid processes, which all develop from separate ossification centers by either endochondral or intramembranous ossification. The sphenoid bone develops from four paired cartilages that are arranged in the following caudocranial sequence: postsphenoid (basisphenoid or hypophysial), presphenoid, alisphenoid and orbitosphenoid ones. The postsphenoid cartilage gives rise to the greater posterior part of the body of sphenoid bone, together with the sella turcica and the hypophysial fossa^5^. On the base of the presphenoid cartilage develop both the sphenoidal plane and the sphenoidal yoke. The alisphenoid cartilage gives origin to both the greater wing—except for its top—and the lateral pterygoid process. The body of sphenoid bone develops from numerous primary ossification centers in its two paired templates that merge together. After the consolidation of these osseous spots the spread of ossification within the presphenoid and postsphenoid (basisphenoid or hypophysial) cartilages is called the presphenoid ossification center and postsphenoid ossification center, respectively. The body of sphenoid bone is gradually pneumatized by the sphenoidal sinus. Furthermore, the sella turcica, which is located on the concave superior surface of body of sphenoid bone, is an important area for the topography of neurovascular and endocrine structures^6,7^.

The present study includes more detailed and novel numerical data regarding the morphometric analysis of the two ossification centers within the presphenoid and postsphenoid cartilages of the body of sphenoid bone in human fetuses based on CT imaging.

In this study, we aimed to determine normative age-specific values for linear, planar and volumetric parameters of the presphenoid and postsphenoid ossification centers in human fetuses and to compute growth dynamics for the analyzed parameters, expressed by best-matched mathematical models.

## Material and methods

The study material comprised 37 human fetuses of Caucasian origin (16 males and 21 females) aged 18–30 weeks of gestation. All experimental protocols were approved by the Bioethics Committee of the Ludwik Rydygier Collegium Medicum in Bydgoszcz (KB 275/2011). The fetuses were obtained from spontaneous miscarriages after receiving parental informed consent in the written form for their use in research. The fetuses were collected before the year 2000 and are currently part of the fetal collection of the Department of Anatomy of the Ludwik Rydygier Collegium Medicum of the Nicolaus Copernicus University in Toruń. All procedures were performed in compliance with the legal regulations in force in Poland and the Donation Corpse program for both adults and fetuses. This study was conducted in accordance with the principles of the Declaration of Helsinki.

The morphometric examinations were conducted between January 1, 2022, and September 30, 2022 at the Department of Anatomy of the Ludwik Rydygier Collegium Medicum of the Nicolaus Copernicus University in Toruń. All fetuses were included in the study based on their explicit morphology and clinical records, and were required to have no conspicuous morphological malformations or developmental abnormalities of the musculoskeletal system. As a result, fetuses with noticeable developmental diseases, such as congenital defects or intrauterine growth retardation, were excluded from the present study. Fetal ages were determined from the crown-rump length and the known date of the beginning of the last maternal menstrual period. Furthermore, only fetuses with a strong correlation (R = 0.98, *p* < 0.001) between the gestational age based on the crown-rump length and that calculated using the last menstruation were included in the study. Table 1 provides the characteristics of the study group, including the fetal age, number and sex of the fetuses studied.

**Table 1:** Age, number and sex of the fetuses studied.

Gestational age (weeks)	Crown-rump length (mm)				Number of fetuses	Sex	
	Mean	SD	Min	Max	N	♂	♀
18	133.33	5.77	130.00	140.00	3	1	2
19	146.50	2.89	143.00	150.00	4	2	2
20	161.00	2.71	159.00	165.00	4	2	2
21	173.67	2.31	171.00	175.00	3	2	1
22	184.67	1.53	183.00	186.00	3	1	2
23	198.67	2.89	197.00	202.00	3	1	2
24	208.00	3.56	205.00	213.00	4	1	3
25	214.00	0.00	214.00	214.00	1	0	1
26	229.00	5.66	225.00	233.00	2	1	1
27	240.33	1.15	239.00	241.00	3	3	0
28	249.50	0.71	249.00	250.00	2	0	2
29	253.00	0.00	253.00	253.00	2	0	2
30	262.67	0.58	262.00	263.00	3	2	1
Total					37	16	21

**Figure 1:**
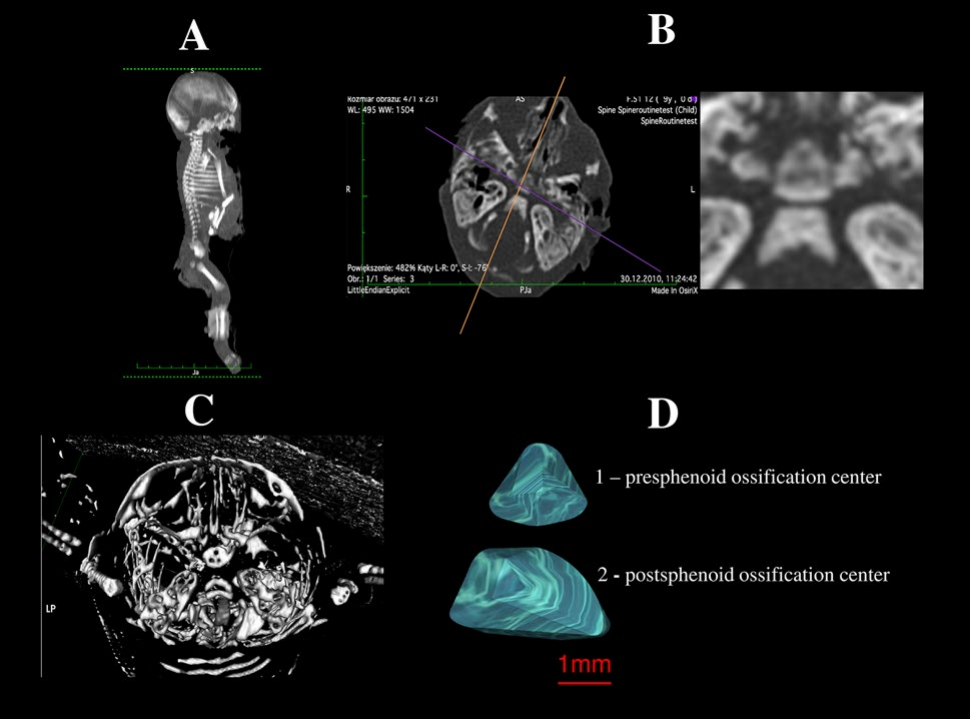
CT of the whole female human fetus aged 26 weeks in the sagittal projection (**A**) with its cranial CT in the transverse projection (**B**), CT reconstruction of the presphenoid and postsphenoid ossification centers using Osirix 3.9 MD (**C**), and volume of the presphenoid (1) and postsphenoid (2) ossification centers (**D**).

With the use of the Siemens-Biograph 128 mCT scanner (Siemens Healthcare GmbH, Erlangen, Germany) located in the Department of Positron Emission Tomography and Molecular Imaging at the Oncology Center of the Ludwik Rydygier Collegium Medicum in Bydgoszcz, the Nicolaus Copernicus University Bydgoszcz, Poland, we acquired scans of fetuses in DICOM format at 0.4 mm intervals, as shown in Fig. 1. It is noteworthy that Osirix 3.9 MD enables precise numerical analysis of linear, planar, and three-dimensional reconstructions of objects studied.

The gray scale of the obtained CT images expressed in Hounsfield units (HU) differed from − 275 to − 134 for a minimum, and from + 1165 to + 1558 for a maximum. Therefore, the window width (WW) varied from 1.404 to 1.692, while the window level (WL) varied from + 463 to + 712. The imaging protocol was specified by the following: mAs—60, kV—80, pitch—0.35, FoV—180, rot. Time—0.5 s., while specifics of the CT data were as follows: slice thickness—0.4 mm, image increment—0.6 mm, and kernel—B45 f.-medium. First the parameters WW and WL were automatically set by the Osirix 3.9 MD software and consecutively they were adjusted by observers for even better imaging of the studied structure.

It should be emphasized that the slice thickness of 0.4 mm may seem to be inadequate for such a precise morphometric analysis. However, the slice thickness of 0.4 mm is just the lowest one for the Siemens-Biograph 128 mCT scanner and is bioethically acceptable for in utero measurements and reflected in the protocol of “SpineRoutineTest (Child)”. As a result, the number of slices visualizing the ossifying sectors of the body of sphenoid bone gradually increased with fetal age. Specifically, the growth of the sagittal diameter of the presphenoid and postsphenoid ossification centers was assessed with the use of 7–11 and 6–9 slices, respectively. The number of slices referring to the transverse diameter was 5–9 for the presphenoid ossification center and 8–11 for the postsphenoid ossification center. By setting the sagittal, vertical and transverse axes at right angles, the sagittal and transverse diameters of both ossification centers were measured in the transverse plane, characterized by the greatest projection surface area.

In spite of the cartilaginous stage of the body of sphenoid bone, both the presphenoid and postsphenoid ossification centers may clearly have been visualized and outlined.

**Figure 2:**
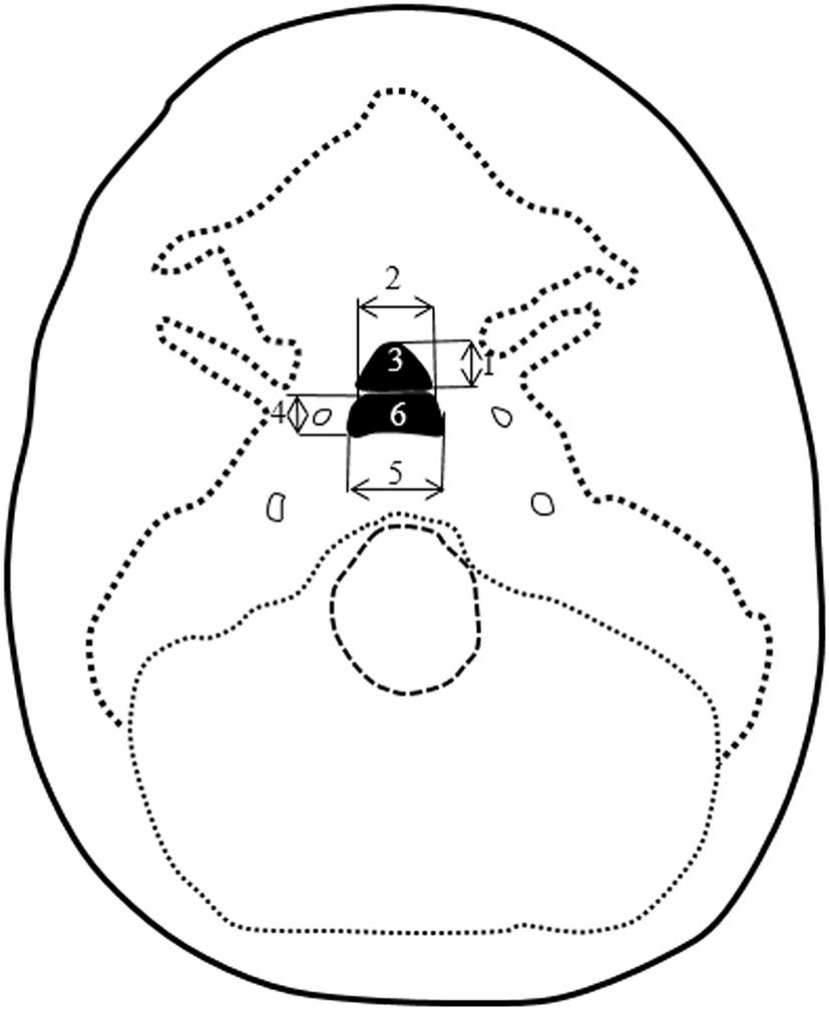
Measurement scheme of ossification centers of sphenoid’s body in the transverse plane: 1—sagittal diameter of presphenoid ossification center, 2—transverse diameter of presphenoid ossification center, 3—projection surface area of presphenoid ossification center, 4—sagittal diameter of postsphenoid ossification center, 5—transverse diameter of postsphenoid ossification center, 6—projection surface area of postsphenoid ossification center.

Measurements of the two ossification centers within the body of sphenoid bone were performed in a specific sequence (Fig. 2). In each fetus, the assessment of linear parameters, projection surface area and volume of the presphenoid and postsphenoid ossification centers was carried out. Both the projection surface area and volume of the presphenoid and postsphenoid ossification centers were semi-automatically calculated by outlining all regions of interest (ROI) in the CT series and then using Osirix 3.9 MD to calculate the ROI volume.

Measurements of both the presphenoid and postsphenoid ossification centers included the following four parameters:

1. sagittal diameter, based on the determined distance between the proximal and distal borderlines of the presphenoid and postsphenoid ossification centers in the transverse plane (Fig. 2);
2. transverse diameter, based on the determined distance between the medial and lateral borderlines of the presphenoid and postsphenoid ossification centers in the transverse plane (Fig. 2);
3. projection surface area, based on the outlined area occupied by the presphenoid and postsphenoid ossification centers in the transverse plane (Fig. 2);
4. volume of the presphenoid and postsphenoid ossification centers, calculated using advanced diagnostic imaging tools for 3D reconstruction, taking into account the position and the absorption of radiation by bone (Fig. 1C).

Numerical data obtained in this study was statistically analyzed using the Statistica 12.5 and PQStat 1.6.2 programs. The distribution of variables was checked using the Shapiro–Wilk test, while homogeneity of variance was analyzed by Fisher’s test.

The developmental dynamics of the analyzed parameters were characterized using linear and non-linear regression analysis. The match between the estimated curves and measurement results was evaluated based on coefficients of determination (R^2^). The relationship between variables was also estimated using the Pearson correlation coefficient (R). Differences were considered statistically significant at *p* < 0.05.

In a continuous effort to minimize measurement and observer bias, all measurements were performed by one researcher (M.B.) and verified by the other examiner (M.G.). The measurements were performed on the same scan that depicted the greatest sagittal and transverse diameters, and projection surface area of the presphenoid and postsphenoid ossification centers. Subsequently, the volume calculations were semi-automatically performed by marking the examined parameter on each slice, the number of which depended on the size and fetal age, thus ranging from 5 to 11. Each measurement was taken three times under the same settings but at different times (in one-day intervals), and then averaged. The inter-observer variation between repeated measurements was assessed by ANOVA.

### Ethical approval

This material has not been published in whole or in part elsewhere. The manuscript is not currently being considered for publication in another journal. The anatomical protocol of the study was accepted by the Bioethics Committee of Ludwik Rydygier Collegium Medicum in Bydgoszcz (KB 275/2011). The fetuses were obtained from spontaneous abortions after parental consent and were from Department of Anatomy of Ludwik Rydygier Collegium Medicum of Nicolaus Copernicus. Everything was in accordance with the legal procedures in force in Poland and in accordance with the program Donation Corpse both adults and fetuses. This study was performed in line with the principles of the Declaration of Helsinki.

## Results

The intra-class correlation coefficients (ICC) were statistically significant ($p < 0.001$) and of excellent reproducibility, as displayed in Table 2.

**Table 2:** Intra-class correlation coefficients (ICC) values.

Parameter	ICC
Sagittal diameter of the presphenoid ossification center	0.998*
Sagittal diameter of the postsphenoid ossification center	0.997*
Transverse diameter of the presphenoid ossification center	0.998*
Transverse diameter of the postsphenoid ossification center	0.999*
Projection surface area of the presphenoid ossification center	0.999*
Projection surface area of the postsphenoid ossification center	0.997*
Volume of the presphenoid ossification center	0.997*
Volume of the postsphenoid ossification center	0.998*

Inter-class correlation coefficients marked with * are statistically significant at *p* < 0.0001.

The arithmetical mean values and standard deviations of the analyzed parameters of the presphenoid and postsphenoid ossification centers in human fetuses at the analyzed gestational ages have been presented in Tables 3 and 4.

**Table 3:** Sagittal and transverse diameters, projection surface area and volume of the presphenoid ossification center in the human fetus.

Gestational age (weeks)	N	Presphenoid ossification center							
		Sagittal diameter (mm)		Transverse diameter (mm)		Projection surface area (mm^2^)		Volume (mm^3^)	
		Mean	SD	Mean	SD	Mean	SD	Mean	SD
18	3	2.84	0.12	2.10	0.06	5.39	0.37	12.22	1.17
19	4	2.97	0.01	2.19	0.03	5.86	0.09	14.37	1.25
20	4	3.12	0.11	2.28	0.07	6.47	0.47	17.29	1.33
21	3	3.26	0.00	2.58	0.02	7.64	0.11	19.32	0.28
22	3	3.48	0.08	2.70	0.03	8.36	0.27	22.44	0.63
23	3	3.60	0.01	2.77	0.03	8.87	0.11	23.54	0.28
24	4	3.71	0.11	2.86	0.04	9.37	0.27	24.79	0.86
25	1	3.92	–	2.93	–	10.00	–	26.40	–
26	2	3.96	0.00	3.07	0.00	10.59	0.00	27.92	0.15
27	3	4.00	0.05	3.20	0.04	11.15	0.28	29.10	0.70
28	2	4.12	0.08	3.25	0.00	11.79	0.24	30.63	0.07
29	2	4.22	0.06	3.25	0.01	12.10	0.19	31.39	0.87
30	3	4.30	0.06	3.27	0.01	12.39	0.18	33.99	1.49

**Table 4:** Sagittal and transverse diameters, projection surface area and volume of the postsphenoid ossification center in the human fetus.

Gestational age (weeks)	N	Postsphenoid ossification center							
		Sagittal diameter (mm)		Transverse diameter (mm)		Projection surface area (mm^2^)		Volume (mm^3^)	
		Mean	SD	Mean	SD	Mean	SD	Mean	SD
18	3	2.15	0.02	3.02	0.08	6.84	0.06	13.82	0.14
19	4	2.30	0.02	3.16	0.05	7.17	0.16	16.30	0.73
20	4	2.50	0.12	3.38	0.08	8.24	0.57	19.12	1.53
21	3	2.73	0.03	3.55	0.03	9.26	0.26	22.15	1.72
22	3	2.88	0.02	3.67	0.01	10.08	0.15	24.82	0.24
23	3	2.94	0.03	3.70	0.01	10.28	0.04	25.44	0.17
24	4	3.07	0.05	3.91	0.17	10.68	0.16	27.00	1.39
25	1	3.20	–	4.12	–	10.87	–	28.81	–
26	2	3.25	0.02	4.14	0.00	11.24	0.52	30.60	0.71
27	3	3.34	0.01	4.27	0.03	12.02	0.28	31.50	0.38
28	2	3.36	0.03	4.34	0.06	12.50	0.07	33.78	0.49
29	2	3.39	0.01	4.44	0.07	12.92	0.07	34.58	0.29
30	3	3.42	0.02	4.50	0.01	13.42	0.12	37.77	2.39

**Figure 3:**
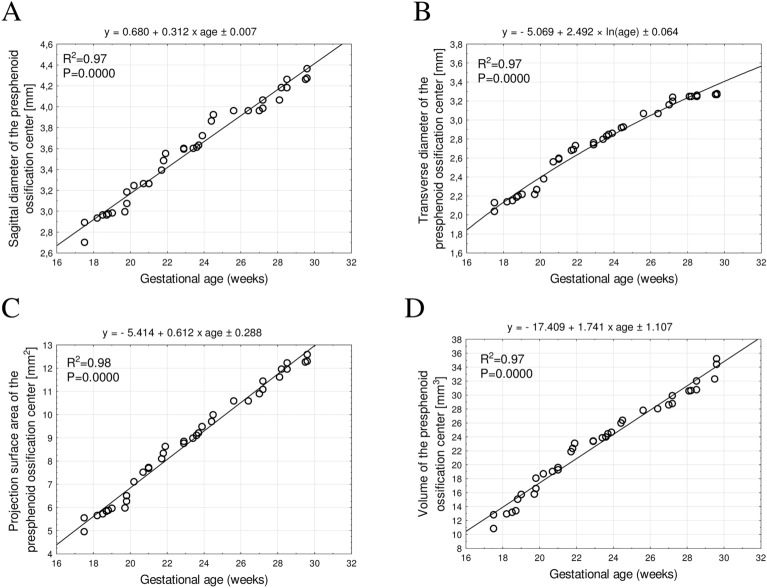
Regression lines for the sagittal diameter (**A**), transverse diameter (**B**), projection surface area (**C**) and volume (**D**) of the presphenoid ossification center.

**Figure 4:**
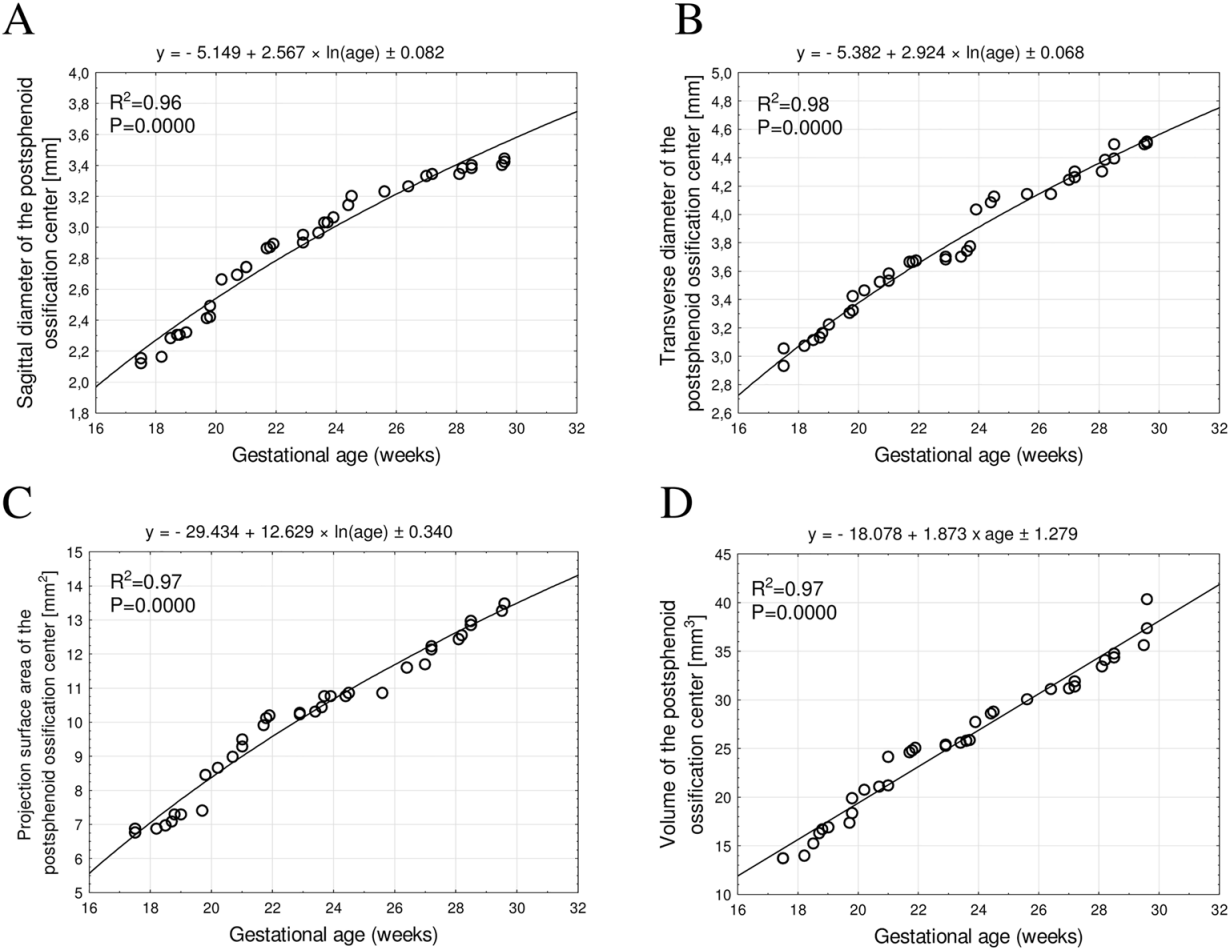
Regression lines for the sagittal diameter (**A**), transverse diameter (**B**), projection surface area (**C**) and volume (**D**) of the postsphenoid ossification center.

For both the presphenoid and postsphenoid ossification centers there were insignificant male–female differences for their sagittal diameters, transverse diameters, projection surface areas and volumes ($p>0.05$). Since the statistical analysis showed no sex differences for the four examined parameters of either ossification center, we aggregated numerical data for both sexes and computed one growth curve for each parameter. The growth patterns for analyzed parameters have been displayed in Figs. 3 and 4.

The sagittal diameter, projection surface area and volume of the presphenoid ossification center, as well as the volume of the postsphenoid ossification center demonstrated linear growths, proportionate to gestational age. In contrast, the transverse diameter of the presphenoid ossification center and the sagittal and transverse diameters, and projection surface area of the postsphenoid ossification center followed natural logarithmic functions.

## Discussion

The body of sphenoid bone is composed of two ontogenetically distinct parts, namely the presphenoid and postsphenoid cartilages, which are separated from each other by the intrasphenoidal synchondrosis. These two parts have different origins and growth rates. From an evolutionary perspective, the posterior part of the cranial base has retained structures present in early mammals, while the anterior part is considered evolutionarily new and has a significant impact on the development of the viscerocranium, which is characteristic of humans^8^.

The whole cranial base presents the endochondral ossification. In the cranial base first appear the orbitosphenoid ossification centers at week 8, the alisphenoid ossification centers at week 9 and the postsphenoid ossification centers at week 11. Afterwards, at week 16 of gestation appear ossification centers in all remaining cartilages of the cranial base^9,10^.

As ossification progresses, most of the chondrocranium is replaced by bone, except for the non-ossified synchondroses which serve as temporary cartilaginous joints between adjacent cranial bones, and eventually are substituted with bony tissue. Like the epiphyseal cartilages in long bones, the synchondroses in the cranial base function as growth plates, which are responsible for the growth of the whole cranium: the cranial base, viscerocranium and calvaria. It is noteworthy that within the sphenoethmoidal and sphenooccipital synchondroses bony tissue is much more deposited on the ethmoid and occipital bones than on the sphenoid bone. This rule refers to both the presphenoid and postsphenoid cartilages in the body of sphenoid bone. The boundary that separates the presphenoid and postsphenoid cartilages is the intrasphenoidal synchondrosis, which ossifies shortly before birth^9,10^.

In the presphenoid cartilage, which constitutes the smaller portion of the body of sphenoid bone, two pairs of ossification centers are typically present: the lateral and medial presphenoid ossification centers^5,11,12^. It is worth mentioning that the lateral presphenoid ossification centers usually ossify earlier than the medial presphenoid ossification centers. Some authors also described accessory ossification centers in the presphenoid cartilage, such as the paired anterior and posterior presphenoid ossification centers, and the single middle presphenoid ossification center. The ongoing aggregation of all these presphenoid ossification centers presents a triangular area known as either the olivary eminence or the anterior foramen. The olivary eminence usually disappears around birth, while the anterior foramen is so small that it should not be mistaken for a sinus or encephalocele ^5^.

In the postsphenoid cartilage, which constitutes the larger portion of the body of sphenoid bone, there are typically four postsphenoid ossification centers: two medial and two lateral ones^5,11–13^. The bilateral medial postsphenoid ossification centers may fuse into a larger middle postsphenoid ossification center. Such variations may explain the different timing for the closure of the craniopharyngeal duct, which remains patent in 0.4% of neonatal skulls during the postnatal period and may lead to craniopharyngioma.

Zhang et al.^4^ found two ossification patterns of the postsphenoid cartilage. In the first pattern, the medial postsphenoid ossification centers fused at an early stage of development (CRL 120–140 mm, 17w0d–18w0d). Contrariwise, in the second pattern, the medial postsphenoid ossification centers remained separate for a longer period and fused at a later stage (CRL 170 mm, 21w0d). These authors also measured the distance between the two most distant points of the postsphenoid ossification center, which reached the mean values: 3.35 ± 0.58 mm in fetuses of CRL 120 mm (17w0d), 4.72 ± 0.48 mm in fetuses of CRL 130 mm (17w5d), 4.41 ± 0.65 mm in fetuses of CRL 140 mm (18w2d), and 4.47 ± 0.69 mm in fetuses of CRL 170 mm (20w6d). For a single postsphenoid ossification center the mean distance was 3.72 ± 0.58 mm, and for a double postsphenoid ossification center the mean distance reached the value of 4.83 ± 0.45 mm. In our study, all examined individuals referred to the gestational age range of 18–30 weeks, with an average transverse diameter of the postsphenoid ossification center ranging from 3.02 ± 0.08 to 4.50 ± 0.01 mm.

The sphenoid bone is of great importance, as variations in its shape are responsible for the variability of the cranial base. Shortening of the body of sphenoid bone in the sagittal dimension may considerably alter the spatial relationship between the cranial base, the viscerocranium and the calvaria, thus leading to the protrusion of viscerocranium^13^.

Since the sphenoid bone plays a crucial role in the morphogenesis of the cranial base, some developmental defects of the sphenoid region may be found in various congenital facial deformities, including craniosynostosis, cleft lip, cleft palate, and Down syndrome^8^.

Utsunomiya et al.^8^ accentuated a minor but still interesting difference in the morphogenesis of the presphenoid and postsphenoid cartilages of the body of sphenoid bone. These authors found changes in the shape of the presphenoid cartilage to take longer than those in the postsphenoid cartilage. The authors suggested that the presphenoid cartilage may be more plastic and so may undergo a stronger reconstruction. Thus, during evolution the presphenoid part had the potential to create specific features of the human face through plasticity and instability. Furthermore, it is suggested that in patients with congenital abnormalities of the viscerocranium, cranial base deformations are more likely to occur in the presphenoid part, including the ethmoid bone, as well.

Utsunomiya et al.^8^ used PCX-KT to measure the distance between the postsphenoid part and the sella turcica in 57 fetuses, starting with week 19. The distances were measured in the sagittal plane and were as follows: 2.33 ± 0.18 mm at week 19, 2.65 ± 0.06 mm at week 20, 2.82 ± 0.22 mm at week 21, 3.05 ± 0.26 mm at week 22, and 3.60 ± 0.31 mm at week 23 week of gestation. In our study, we did not measure the sagittal dimension between the postsphenoid part and the sella turcica. Instead, we measured the sagittal and transverse diameters of the postsphenoid ossification center in the transverse plane. At the age range of 18–23 weeks, the mean sagittal and transverse diameters of the postsphenoid ossification centers ranged from 2.30 ± 0.02 to 2.94 ± 0.03 mm, and from 3.16 ± 0.05 to 3.70 ± 0.01 mm, respectively.

To our best knowledge, the present article is the first to concentrate on the morphometric analysis of the presphenoid and postsphenoid ossification centers in human fetuses using CT and their growth dynamics. Our study involved mathematical models, so as to describe the growth of the presphenoid and postsphenoid ossification centers within the body of sphenoid bone. We found the sagittal diameter, projection surface area, and volume of the presphenoid ossification center, as well as the volume of the postsphenoid ossification center to follow proportionately to gestational age in weeks, thus indicating a commensurate growth between 18 and 30 weeks of gestation. On the other hand, the transverse diameter of the presphenoid ossification center, as well as the sagittal and transverse diameters and projection surface area of the postsphenoid ossification center followed natural logarithmic functions, with gestational ages expressed in weeks. The apposite selection of the best-fit function results from the highest value of coefficient of determination R^2^, which effectively illustrates the growth of the studied parameter and the degree of fit to the function. In our study, the highest coefficients of determination ranged from 0.96 to 0.98, indicating an excellent fit of the growth model. The linear growth is directly proportionate to fetal age, while the natural logarithmic growth is marked by initial intensive growth, followed by a gradual deceleration of growth with fetal age.

In our earlier study about the development of the ossification centers of the lateral and basilar parts of the occipital bone, their growth dynamics followed linear functions with gestational age^14^.

Levaillanto and Mabille^15^ compared the images of the sphenoid bone in fetuses acquired by 3D ultrasound and CT, and found the images obtained using both techniques to be alike. Therefore, results obtained from the morphometric measurements of ossification centers with the use of CT may allow for the more accurate diagnostics of developmental disorders and may enable further research of developmental processes and biometric relationships.

The present paper is the first account to precisely describe linear, planar and volumetric parameters of both the presphenoid and postsphenoid ossification centers with modelling their growth dynamics in fetuses aged 18–30 weeks.

There is still little information in the professional literature about the quantitative anatomy of the fetal skeletal system at specific gestational weeks with the use of CT. Furthermore, there are currently no reports in the medical literature concerning any dimensions of the presphenoid and postsphenoid ossification centers in human fetuses that limits a comprehensive discussion on this topic. Therefore, the findings obtained in the present study may contribute to a better understanding of the growing fetal skeleton, bringing new numerical information regarding its diagnosis and development. Knowledge about the growth of individual cranial bones in human fetuses may be conducive in such numerous fields as anatomy, anthropology, forensic medicine, orthodontics, radiology, obstetrics, pediatrics, orthopedics and reconstruction surgery.

Our numerical data on the presphenoid and postsphenoid ossification centers may be of potential relevance in monitoring the normal fetal growth and screening for congenital disorders in the fetus.

The main limitation of this study was the relatively narrow gestational age group, which ranged from 18 to 30 weeks, and the number of human fetuses reduced to 37.

## Conclusions

The presphenoid ossification center presents linear growths in its sagittal diameter, projection surface area, and volume, and natural logarithmic growth in its transverse diameter. The postsphenoid ossification center displays natural logarithmic growths in its sagittal and transverse diameters, as well as its projection surface area, and a linear growth in its volume. The linear growth manifests a consistent increase in values of morphometric parameters with advanced fetal age, while the natural logarithmic growth demonstrates an initial rapid increase in values of morphometric parameters that then slows down with advanced fetal age. The obtained morphometric data of the presphenoid and postsphenoid ossification centers may serve as age-specific reference intervals, aiding in the estimation of gestational ages and the ultrasonic diagnosis of congenital defects. Further research on the growth and morphometric characteristics of these ossification centers is warranted to enhance understanding of their development and potential clinical significance.

## Data Availability

Any additional data supporting this study are available from the corresponding author (M.G.) upon reasonable request.

## References

[1] Duarte WR, Shibata T, Takenaga K, Takahashi E, Kubota K, Ohya K (2013). S100A4: A novel negative regulator of mineralization and osteoblast differentiation. J. Bone Miner. Res..

[2] Grzesiakowska U, Tacikowska M (2005). Imaging of the skull base. Pol. J. Radiol..

[3] Krakow D, Lachman RS, Rimoin DL (2009). Guidelines for the prenatal diagnosis of fetal skeletal dysplasias. Genet. Med..

[4] Zhang Q, Wang H, Udagawa J, Otani H (2011). Morphological and morphometric study on sphenoid and basioccipital ossification in normal human fetuses. Congenit. Anom. (Kyoto).

[5] Nemzek WR, Brodie HA, Hecht ST, Chong BW, Babcook CJ, Seibert JA (2000). MR, CT, and plain film imaging of the developing skull base in fetal specimens. AJNR Am. J. Neuroradiol..

[6] Yamamoto M, Abe H, Hirouchi H, Sato M, Murakami G, Rodríguez-Vázquez JF (2021). Development of the cartilaginous connecting apparatuses in the fetal sphenoid, with a focus on the alar proces. PLoS ONE.

[7] Budu V, Mogoantă CA, Fănuţă B, Bulescu I (2013). The anatomical relations of the sphenoid sinus and their implications in sphenoid endoscopic surgery. Rom. J. Morphol. Embryol..

[8] Utsunomiya N, Katsube M, Yamaguchi Y, Yoneyama A, Morimoto N, Yamada S (2022). The first 3D analysis of the sphenoid morphogenesis during the human embryonic period. Sci. Rep..

[9] Cendekiawan T, Wong RWK, Rabie ABM (2010). Relationships between cranial base synchondroses and craniofacial development: A Review. Open. Anat. J..

[10] Perkowski K, Szpinda-Barczyńska A, Kamiński K (2020). Growth of the cranial base and its influence on the position of the maxilla and mandible—A literature review. Forum Ortod..

[11] Mano N, Wood B, Oladipupo L, Reynolds R, Taylor J, Durham E (2021). The chondrocranial key: Fetal and perinatal morphogenesis of the sphenoid bone in primates. Vertebr. Zool..

[12] Mehemed TH, Fushimi Y, Okada T, Kanagaki M, Yamamoto A, Okada T (2016). MR imaging of the pituitary gland and postsphenoid ossification in fetal specimens. Am. J. Neuroradiol..

[13] Morimoto N, Ogihara N, Katayama K, Shiota K (2008). Three-dimensional ontogenetic shape changes in the human cranium during the fetal period. J. Anat..

[14] Grzonkowska M, Baumgart M, Badura M, Wiśniewski M, Lisiecki J, Szpinda M (2021). Quantitative anatomy of primary ossification centres of the lateral and basilar parts of the occipital bone in the human foetus. Folia Morphol. (Warsz).

[15] Levaillanto JM, Mabille M (2008). Fetal sphenoid bone: imaging using three-dimensional ultrasound and computed tomography. Ultrasound Obstet. Gynecol..

